# Health-care cost reduction resulting from primary-care allergy testing in children in Italy

**DOI:** 10.1186/1824-7288-36-61

**Published:** 2010-09-13

**Authors:** Niklas Zethraeus, Carl Johan Petersson, Massimiliano Dozzi, Magnus P Borres, Giulio Vignati, Alessandro Fiocchi

**Affiliations:** 1 Medical Management Centre (MMC), Institution for Learning, Informatics, Management and Ethics (LIME), Karolinska Institutet, Stockholm, Sweden; 2 Centre for Health Economics, Stockholm School of Economics, Stockholm, Sweden; 3 Medical Department, Phadia AB, Uppsala, Sweden; 4 Ambulatorio Pediatrico, P.ZA DEL POPOLO 3, Magenta, Italy; 5 Centro Malattie Endocrine e Metaboliche, Ospedale G.Fornaroli Magenta, Italy; 6 Child & Maternal Medicine, The Melloni Hospital, Milan, Italy

## Abstract

**Background:**

Allergy places a considerable cost burden on society. Specific immunoglobulin E (spIgE) testing may improve the management of allergy patients. There is therefore a reason to quantify the economic consequences of the use of spIgE testing in the diagnosis of allergic conditions.

**Methods:**

The expected costs of spIgE testing versus no-testing were calculated using a clinical decision model based on a prospective clinical trial performed in primary care.

**Results:**

The expected costs per patient over 2 years decreased from 802 euros in the "no-test strategy" to 560 euros in the spIgE "test strategy". Cost savings persisted even after assumptions about the prevalence of allergy and the prices of medications were changed. The "test strategy" increased the percentage of patients correctly diagnosed from 54 to 87%.

**Conclusions:**

spIgE testing of children with respiratory and/or skin problems in primary care in Italy reduces overall costs to society. These cost savings mostly result from a reduction in the use of medications, particularly corticosteroids. The study indicates that spIgE testing of all children with respiratory and/or skin symptoms would be a cost-effective strategy.

## Background

Over the past 40 years, the prevalence of atopic disease has increased, particularly in Western, industrialised countries; most of the allergy-related morbidity associated with the respiratory system is accounted for by asthma and allergic rhinitis [1]. Importantly, these conditions substantially affect patients' quality of life [2-4] placing a large burden on society in terms of both direct medical expenditure and decreased productivity [5,6]. In the U.S., the estimated annual cost for asthma is US$14 billion in direct and indirect costs [7]. The total per-person annual costs of asthma in this country average $4912.00, with direct and indirect costs accounting for $3180 (65%) and $1732 (35%), respectively. The largest components within direct costs are pharmaceuticals [$1605 (50%)], hospital admissions [$463 (15%)] and non-emergency department ambulatory visits [$342 (11%)] [8]. Such burden, reduced by an appropriate diagnostic and screening process for allergy aimed to increase the appropriateness of referrals from primary care to the specialist level[9,10], is paid for at the social level either directly by managed care systems or indirectly by households and businesses.

In the managed-care environment of North America [11], stretched healthcare budgets have to meet the growing needs and demands of the population as well as the increasing costs of new drugs, medical devices and diagnostic tests. Limited health care resources have to be used efficiently in order to maximise health outcomes. Assessment of the costs and benefits of different ways of allocating resources assists decisions for enhancing efficiency. Economic evaluation plays an important role in pricing and reimbursement decisions made by agencies such as the Dental and Pharmaceutical Benefits Agency (TLV) in Sweden and provides important input for bodies producing guidance for clinical decisions such as the National Institute for Health and Clinical Excellence (NICE) in England and Wales and the guideline committee of the National Board of Health and Welfare in Sweden.

In managed healthcare, where GP reviews of individual allergy and asthma patients form the basis for epidemiological and economic reports [12], selection of an appropriate allergy diagnostic methodology can play a significant economic role.

There is currently no all-purpose (or all-setting) allergy diagnostic method or standard [13]. As a case in point, a study conducted in The Netherlands showed that patients with asthma were highly unlikely to be told whether their condition was allergic or non-allergic, and very few patients had ever seen an allergist or received any allergy test [14]. Improved diagnosis and management of patients with allergy symptoms in primary care is therefore essential, but despite the known benefits of allergy testing, and although it may account for only a small proportion of total, direct healthcare costs [15], some physicians still perceive testing as an unnecessary expense.

The present study was designed as a cost analysis of specific immunoglobulin E (spIgE) testing in primary care compared with no-testing of children with respiratory and/or skin symptoms by means of a clinical decision model based on a clinical trial [16]. The cost comparison was made from a managed healthcare perspective and applied in a European primary care setting, but could easily apply as a template for U.S. - derived data. The percentage of patients correctly diagnosed was estimated for the two strategies compared here.

## Methods

### The clinical trial on which this study was based

The basis for the economic evaluation and the clinical decision model was a non-randomised clinical trial [16]. This trial investigated the impact of adding spIgE testing of blood on the current management by primary-care physicians of children with respiratory and/or skin symptoms in 721 subjects in Spain and Italy. It was a prospective one-visit study with no follow-up. All participants were informed about the study and signed an informed consent before participating in the study. The protocol was reviewed and approved by the Comité Étic d'Investigació Clinica de la Fundació Jordi Gol i Gurina, Barcelona on February 28th 2002 (prior to the initiation of the study).

The primary objective of that study was to evaluate the effect of the spIgE test on the frequency of prescriptions for antihistamines, corticosteroids and bronchodilators, and the frequency of advice to avoid allergens. Secondary objectives were to evaluate the frequency of referral to an allergist at hospital, of repeat appointments in primary care, and of diagnostic tests other than the study tests. The study had a prospective before/after design and consisted of two parts. In Part I the results of the spIgE tests were not revealed to the clinicians before their diagnosis of the patients as allergic, non-allergic or uncertain. In Part II the result of the spIgE test was known to the diagnosing physician. Between Parts I and II physicians were given a short course on blood testing for IgE-mediated allergy, conducted by personnel with special training in the clinical application of *in-vitro *allergy testing [16]. The conclusions were that the use of specific IgE antibody determinations improves the clinical management of patients with allergy-related symptoms in primary care, allowing advice to be given on specific allergen avoidance.

The "test strategy" of the present study involved the same clinicians, with a new patient cohort. spIgE antibodies were measured in each patient's blood using the ImmunoCAP^® ^analytical system (Phadia AB, Uppsala, Sweden) including either Phadiatop Infant^® ^(0-5 years of age) or Phadiatop^® ^and food mix (fx5e) for patients above the age of 5 years. In accordance with, the tests the patients were classified as spIgE-positive or spIgE-negative.

The Italian part of the study included 344 patients in or referred by ten primary care centres; ten pediatricians participated. Patient inclusion criteria were age 0-14 years and the presence of at least one of the following respiratory and skin symptoms at any time during the previous year: wheezing in the chest, shortness of breath not correlated with physical exercise, long-standing cough, itchy throat, recurrent symptoms of cold, recurrent sneezing, runny or blocked nose when not having a cold, eczema, urticaria and angioedema. Patients who had had an allergy test (skin prick or spIgE) during the last 12 months, the results of which were known by the clinician, were excluded, as were patients infected with human immunodeficiency virus and those with a history of hepatitis. 194 patients participated in Part I (mean age 5 years; 57% females; 54% below the age of 5 years), while 150 patients were included in Part II (mean age 6 years; 55% females; 45% < 5 years). No significant differences were found in the distribution of gender and symptoms between the patient groups in Part I and II.

### Different kinds of economic evaluations

An economic evaluation may be classified as cost-benefit analysis, cost-effectiveness analysis or cost-minimization analysis. In a cost-benefit analysis, benefits and costs are measured in monetary units and a treatment is called cost-effective if the benefits exceed the costs. In a cost-effectiveness analysis costs are measured in monetary units and health effects in non-monetary units such as gained quality-adjusted life-years (QALYs). A treatment is defined as cost-effective (compared with an alternative) if the societal value exceeds the costs of a gained QALY [17]. If the two treatment alternatives under consideration achieve the same health effect or are broadly equivalent, a straight cost comparison in a cost-minimization analysis is sufficient. In this clinical study no QALYs of direct relevance for the patient were available: the only outcome measure was whether a patient was correctly classified as being allergic or not. If the "test strategy" found more patients to be correctly diagnosed, it is reasonable to assume that spIgE testing also improves quality of life, so that if the total cost of this strategy is equal to or less than that of no testing, spIgE testing may be denoted cost-effective.

### The clinical decision model and the calculation of expected costs and outcomes

**Figure 1:**
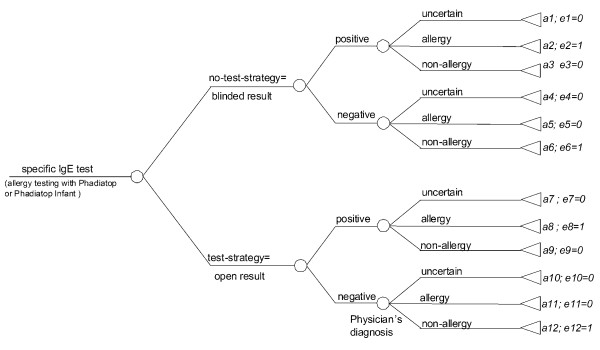
**The clinical decision model with different final endpoints and outcomes**. Twelve possible diagnostic categories *a*1 to *a*12 are shown. The associated probability *p *of a patient falling into each category is shown in Table 2, as well as the expected cost per category over 1 and 2 years from initial visit. The outcome, i.e. degree of agreement *e *between initial allergy test result and the diagnosis assigned (*e *= 1 denotes agreement) is shown in the figure.

The expected costs and outcomes for the two strategies, the "no-test strategy" and the *"*test strategy", were calculated using a clinical decision model (Figure 1) that reflects 12 possible pathways or diagnostic categories, *a*1 to *a*12, and outcomes (*e*1-*e*12)[18]. The calculations refer to an identified child in primary care with respiratory and/or skin symptoms (Table 1). The path probabilities (*p*1-*p*12, Table 2) of the various final endpoints are based on the actual distribution of patients in the clinical trial in Italy. For example, 60% (7.7+52.3) of all patients in the "test strategy" were diagnosed allergic, whereas the corresponding figure was 70% in the "no-test strategy". The fraction of patients having a positive spIgE test-result (prevalence of allergic sensitization) is assumed to be 0.55. This is based on the fraction of Italian patients, in Part I and II, which were classified spIgE positive (190/344). Given the test result, patients were diagnosed into "uncertain", "allergic" or "non-allergic" based on the actual distribution of Italian patients in the clinical trial (Table 2, Figure 1). For example, the share of patients in the "test strategy" with a positive test result that were diagnosed allergic was set to 0.95 (87/92, Table 2). The corresponding fraction of patients in the "no-test strategy" with a positive test result that were diagnosed allergic was set to 0.81 (79/98).

**Table 1:** Costs, in the 1^st ^and 2^nd ^year and their sum after the initial primary care consultation, of use of medications and of subsequent physician visits and the average number (*n*) of courses of medication, further visits and tests experienced by a patient in each diagnostic category

Cost items	Cost (€) year		Mean number of episodes in categories *a1 *to *a12*												
	
	***1***^***st***^	***2***^***nd***^	*sum*	*a1*	*a2*	*a3*	*a4*	*a5*	*a6*	*a7*	*a8*	*a9*	*a10*	*a11*	*a12*
Initial allergy test	12	12	24	0	0	0	0	0	0	1(0)^e^	1(0)	1(0)	1(0)	1(0)	1(0)
Subsequent sIgE tests	8.8	8.5	17.3	0	0	0	0	0	0	10(0)	10(0)	10(0)	0(0)	0(0)	0(0)
Antihistamine use	182^a^	177	359	0.56	0.44	0.60	0.43	0.43	0.19	0	0.62	0.50	0	0.20	0.09
Corticosteroids use	730^b^	709	1439	0.44	0.38	0.40	0.48	0.37	0.10	0	0.23	0.25	0.67	0	0.11
Bronchodilators use	64^c^	62	126	0.11	0.28	0.40	0.33	0.31	0.05	0	0.10	0.25	0	0.10	0.04
Visit Primary care	65	63	128	0.56	0.63	0.60	0.52	0.52	0.29	0	0.60	1	0.33	0.20	0.38
Visit to allergist	65	63	128	0.11	0.24	0	0	0.17	0.05	0	0.32	0.50	0	0.10	0.16

^a ^Based on 1 tablet ceterizin per day for 1 year

^b ^Based on fluticason, 125-250 mg, 4 puffs/day

^c ^Based on salbutamol and fenoterol, 100 mg, 4 puffs/day

^d^Costs in 2^*nd *^year were discounted to correspond to 1st year (rate:3%)

^e^No tests were performed during 2^nd ^year

**Table 2:** Strategy, IgE results, physician's diagnosis, prevalences, frequencies, probabilities and costs in the twelve possible diagnostics categories, *a1 *to *a12.*

Category (no. obs)		Strategy	Initial IgE test	Physcian's diagnosis	^**1**^**Prevalence**	Observed frequence	^**2**^**Probability (*p*)**	^**3**^**Expected category cost (€)**		
								
								year 1	^**4**^**year 2**	year 1&2
*a1*	(9)	no-test	pos	uncertain	0,55	0,09	0,050	24	23	47
*a2*	(79)	no-test	pos	allergy	0,55	0,81	0,446	192	186	377
*a3*	(10)	no-test	pos	no-allergy	0,55	0,10	0,055	26	25	52
*a4*	(21)	no-test	neg	uncertain	0,45	0,22	0,099	48	46	94
*a5*	(54)	no-test	neg	allergy	0,45	0,56	0,252	105	102	206
*a6*	(21)	no-test	neg	no-allergy	0,45	0,22	0,099	13	13	26
*a7*	(1)	test	pos	uncertain	0,55	0,01	0,006	1	0	1
*a8*	(87)	test	pos	allergy	0,55	0,95	0,523	232	175	408
*a9*	(4)	test	pos	no-allergy	0,55	0,04	0,022	12	9	21
*a10*	(3)	test	neg	uncertain	0,45	0,05	0,023	12	12	24
*a11*	(10)	test	neg	allergy	0,45	0,17	0,077	6	5	10
*a12*	(45)	test	neg	no-allergy	0,45	0,78	0,351	51	46	97

^1^frequence of positive results during screening, i.e. positive results in Phadiatop/Padiatop Infant

^2^calculated from the prevalence and observed frequence of being in category

^3^calculated as the sum of costs for medications, physician visits and allergy tests adjusted for observed episodes per patient on pathway as given in Table 1 and probability of being in category.

^4^costs in 2^nd ^year were discounted to correspond to 1^st ^year (rate:3%)

### Definition and estimation of costs and outcomes

The following direct costs were included, based on the clinical study [16]: medicines (antihistamines, corticosteroids and bronchodilators), physician visits in primary and specialist care at hospital (allergist), and allergy tests. The educational cost of training and informing about the principles of spIgE blood testing was not included in the costs because it was considered to be protocol-related and may also be expected to be included in standard medical training. In clinical practice this cost is probably rather low. The time horizon in the cost-minimization analysis was set to 2 years. The reason for this is that the results from spIgE measurements would provide valuable clinical information for some time after the first year. It was assumed that the number of medication episodes and the number of physician visits to primary and specialist care were the same in year 2 as in year 1. No allergy tests were assumed to take place in the second year. Costs in the second year were discounted to present value using a rate of 3% [17]. All costs are expressed in euros at 2004 prices.

To estimate the costs for each pathway (*a*1 to *a*12, Figure 1), the cost items; mean number of medication episodes, physician visits and tests were multiplied by the probability of being in category and the figure of each cost item respectively and eventually summarized together (Tables 1 and 2). All quantities were based on the findings in the clinical trial [16]. If an initial spIgE test was positive, ten additional spIgE tests were assumed to be needed during the first year.

The annual costs per patient for allergy medications are based on the prices from pharmacies in Italy, and were calculated assuming full medication intake of antihistamines (€182 annually; based on ceterezin, 1 tablet/day and price €9.95 per package of 20 tablets), corticosteroids (€730; based on fluticasone, 125-250 mg, 4 puffs/day, and price €40-80 per package of 120 puffs) and bronchodilators (€64; based on salbutamol and fenoterol, 100 mg, 4 puffs/day, and price €4.55-5.95 per package of 120 puffs). The costs per physician and specialist visit in primary care and hospital were set to €65, based on figures from the Italian national health system, the Servizio Sanitario Nazionale. The Servizio Sanitario Nazionale reimbursement system for allergy tests charges €12 for a general atopy test such as Phadiatop^®^, and €8.80 for a spIgE test.

The outcome measure was defined as the fraction of patients who were "correctly" diagnosed as having or not having allergy, defined as the agreement between allergy test result and physicians' allergy classifications. Each outcome (*e*1-*e*12) in Figure 1 was scored 1 (agreement) or 0 (non-agreement).

### Sensitivity analysis

Different prevalences of allergy were reported in Parts I (51% positive) and II (61% positive) in the clinical trial, and we took the overall mean, 0.55, as the prevalence in the initial analysis. However, we assessed the role of prevalence in determining costs in a sensitivity analysis by varying the prevalence between 0 and 100%. A second sensitivity analysis evaluated the impact on expected costs of separately changing the costs of medication, physician visits and allergy tests: each cost was increased or decreased by 50%.

## Results

The expected cost for the "test strategy" was lower compared with the "no-test strategy". The total (discounted) cost-savings over a 2-year period was €242 (Table 3). The cost savings associated with the "test strategy" were primarily the result of changes in the prescription of allergy medication. Over the 2-year period, the medication costs decreased by €305 in the "test strategy" compared with the "no-test strategy". The costs for primary-care physician visits were similar for the two strategies. The "test strategy" also implied cost-savings the first year estimated at €93. Further, the "test strategy" yielded a percentage of patients correctly diagnosed as 87% [derived from Figure 1, nodes *a*8 and *a*12, as (0.55*0.95 = 52.3) + (0.45*0.78 = 35) = 87.3%] as opposed to the no-test strategy, where the yield was 54% [(0.55*0.81 = 44.5 + 0.45*0.22 = 9.9) = 54.4%].

**Table 3:** Expected average costs (€) per patient for the "no-test" and "test strategy".

	Strategy		Difference
	
	No-test	Test	
Total costs year 1	407,07	314,17	-92,90
*allergy tests*	0	61	60,51
*medications*	360,69	205,92	-154,77
*physician visits*	46,38	47,74	1,36
Total costs year 2	395,22	246,27	-148,95
*allergy tests*	0	0	0,00
*medications*	350,19	199,92	-150,26
*physician visits*	45,03	46,35	1,32
Total costs year 1 and 2	802,29	560,44	-241,85
*allergy tests*	0,00	60,51	60,51
*medications*	710,88	405,85	-305,03
*physician visits*	91,41	94,08	2,67

**Figure 2:**
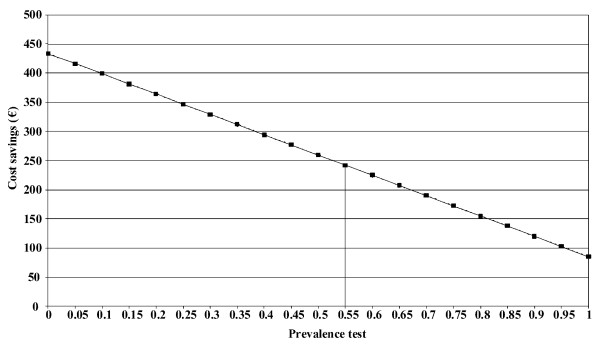
**Cost savings (€) for different assumptions for the prevalence of patients with allergy as judged by the spIgE test**.

The sensitivity analysis showed that the cost-savings decrease with a higher prevalence of allergy, or conversely the lower the prevalence, the higher the cost-savings (Figure 2). The cost savings varied between €220 and €260 if using the prevalence figures of 0.61 and 0.51 respectively, which reflect the two Italian patient groups in Part II and I in the clinical study. The cost-savings also persisted after changing the costs of medication, visits and allergy tests.

## Discussion

The results show that spIgE testing of children with respiratory and/or skin problems on visiting the primary care physician is a cost-saving strategy. The savings are largely due to the reduced need for medications such as antihistamines, bronchodilators, and particularly corticosteroids. Initial spIgE testing decreased the average number of medication episodes over 2 years from 0.85 to 0.76 (11%) for antihistamines, from 0.54 to 0.16 (70%) for bronchodilators, and from 0.73 to 0.36 (51%) for corticosteroids. As shown in Table 2, the proportion of uncertain and false-positive diagnoses decreased substantially in Part II compared with Part I. This indicates that diagnostic categorization could be associated with more appropriate treatment. However, for medication with bronchodilators, normally prescribed for wheezing independently of spIgE, the large decrease was a little unexpected. One explanation could be that an uncertain diagnosis increase the likelihood of overuse of drugs, in this case bronchodilators. Another explanation could be that there was a slightly lower prevalence of wheeze-related symptoms in Part II than in Part I (48% vs 40%, data not shown). Reduced use of corticosteroids accounted for 87% of the total savings in allergy medication.

The spIgE testing also increased the fraction of patients who were correctly diagnosed. Earlier studies[19-21] have shown similar outcomes regarding diagnosis. When patients with allergy-like symptoms were diagnosed on the basis of case history and physical examination alone, only 30-65% received a correct diagnosis, a figure that increased to 85-97% if allergy tests were used.

A full economic evaluation would require a health outcome measure of direct relevance to the patient rather than the proxy measure used here, but the higher fraction of correctly diagnosed patients suggests that patients' health outcomes and therefore quality of life were increased by the test strategy.

The sensitivity analysis showed that spIgE testing saves costs even if the costs of medications were lowered by 50%. A notional 50% decrease in the price of corticosteroids decreased total cost savings by 55%. These results are consistent with studies showing that the cost of medication, in particular corticosteroids, is more significant than hospitalization costs in patients with allergy [22,23]. The sensitivity analysis further showed that the cost savings varied inversely with the prevalence of allergy. Given a negative spIgE test in the no-test strategy, physicians more frequently made the wrong diagnosis than when the spIgE test was positive. The test strategy increased the percentage of patients correctly diagnosed from 22 to 78% for a spIgE negative patient population (Figure 1, nodes *a*4 and *a*12), and from 81 to 95% for a spIgE positive patient population (nodes *a*2 and *a*7).

The cost analysis was based on clinical data obtained from an unselected primary care population, so the external validity of the results should be fair. Some limitations of the clinical study should, however, be pointed out. *First*, only costs for health care were included. Ideally, a societal perspective should be adopted which also includes indirect costs such as lost productivity due to reduced working capacity. European studies of patients with asthma have shown that, unlike the North-American situation [8], the indirect costs are equal to or even higher than the direct costs [24,25]. In the case of children, parents are hindered in their work situations due to the need to accompany their child to the primary care physician. This approach must also include costs of actions taken by the family in avoiding allergens. *Second*, the clinical study was a one-visit study with no follow-up. Ideally, we would have preferred to follow the patients during, say, 2 years after the first physician visit in primary care. Furthermore, the results from this study reflect a common patient seeking help in the primary care setting. Differentiating into different allergy types and/or age classes would demand a much larger study population than that used in our study. *Third*, no outcome measure of direct relevance for the patient, such as a measure of the quality of life, was available.

The findings of this study provide support for evidence-based recommendations for allergy testing in children. The guidelines state that allergy testing is an important prerequisite for early identification of infants at increased risk for later development of allergic diseases, and for specific allergy treatment [26]. In addition, it is recommended that all children with persistent, recurrent or severe allergic symptoms, or who need continuous treatment, should be tested for allergy, regardless of the age of the child. To improve decisions on the efficient allocation of resources for allergy testing in primary care further, randomized studies are needed which examine the costs and quality of life in a naturalistic setting.

Our findings support the cost-effectiveness of spIgE measurement for the diagnosis and management of allergy in clinical practice. It has been suggested that early diagnosis followed by appropriate treatment may interrupt or improve the progression of allergic disease [27]. Implementing avoidance, which can reduce the need for medications [28] is impractical if specific allergens have not been identified. If specific IgE results are positive, the pediatrician can use those results to recommend avoidance of specific allergens or use pharmacotherapy to prevent or treat symptoms. On the contrary, negative results can prevent unnecessary trials of allergy medication, direct further diagnostic efforts, and spare the inconvenience of avoidance for patients who would not benefit [19]. A negative specific IgE result would prompt the pediatrician to look for other causes of symptoms. By providing an accurate definitive diagnosis, specific IgE testing supports early, appropriate, and targeted therapy, greater patient satisfaction, and better control of costs. These data may prove the basis for further analysis in various national contexts with the progress of managed care to improve allergy and asthma outcomes in children.

## Competing interests

CJP and MPB are Phadia AB employees. AF has received research support from Phadia AB. NZ, MD and GV have declared that they have no conflict of interest.

## Authors' contributions

All the authors contributed to the development of the study protocol. The manuscript was drafted by NZ, revised by CJP and AF, and read and approved by all authors.
